# Case of recalcitrant amyopathic dermatomyositis treated with upadacitinib

**DOI:** 10.1016/j.jdcr.2024.08.047

**Published:** 2024-10-01

**Authors:** Albert E. Zhou, Maria Katsetos, Jun Lu

**Affiliations:** Department of Dermatology, University of Connecticut, Farmington, Connecticut

**Keywords:** autoimmune muscle diseases, dermatomyositis, DM, immunopathology, interferon signaling, JAK inhibitors, JAK/STAT pathway, tofacitinib, treatment resistance, upadacitinib

*To the Editor:* We read with great interest the article by Sohn *et al* regarding the successful treatment of refractory amyopathic dermatomyositis (DM) using upadacitinib.^1^ We would like to extend the discussion by highlighting another case where upadacitinib shows promise for clinically amyopathic dermatomyositis (CADM), particularly in patients who have lost effectiveness with other JAK/janus kinase/signal transducer and activator of transcription proteins (STAT) inhibitors.

A 67-year-old female presented with an 8-year history of an extensive, chronic photosensitive rash associated with extreme pruritus. Since her biopsy in 2015 indicated interface dermatitis, she was initially diagnosed with cutaneous lupus erythematosus and treated with hydroxychloroquine and prednisone during flares with minimal improvement. On exam, she had a heliotrope rash, periorbital edema, Gottron’s papules, and Gottron’s erythema on the dorsum of her hands with periungual telangiectasia, and extensive poikiloderma involving the scalp, face, chest, back, bilateral upper extremities, and lateral thighs (Fig 1, *A*). She had clinically normal muscle strength, denied subjective weakness, and lacked muscle atrophy on magnetic resonance imaging. Workup was negative for malignancy but positive for anti-TIF1 gamma antibody and antinuclear antibody titer dilution of 1:160. Creatine kinase and aldolase were within normal limits. By clinicopathological correlation, she was diagnosed with CADM.

**Fig 1:**
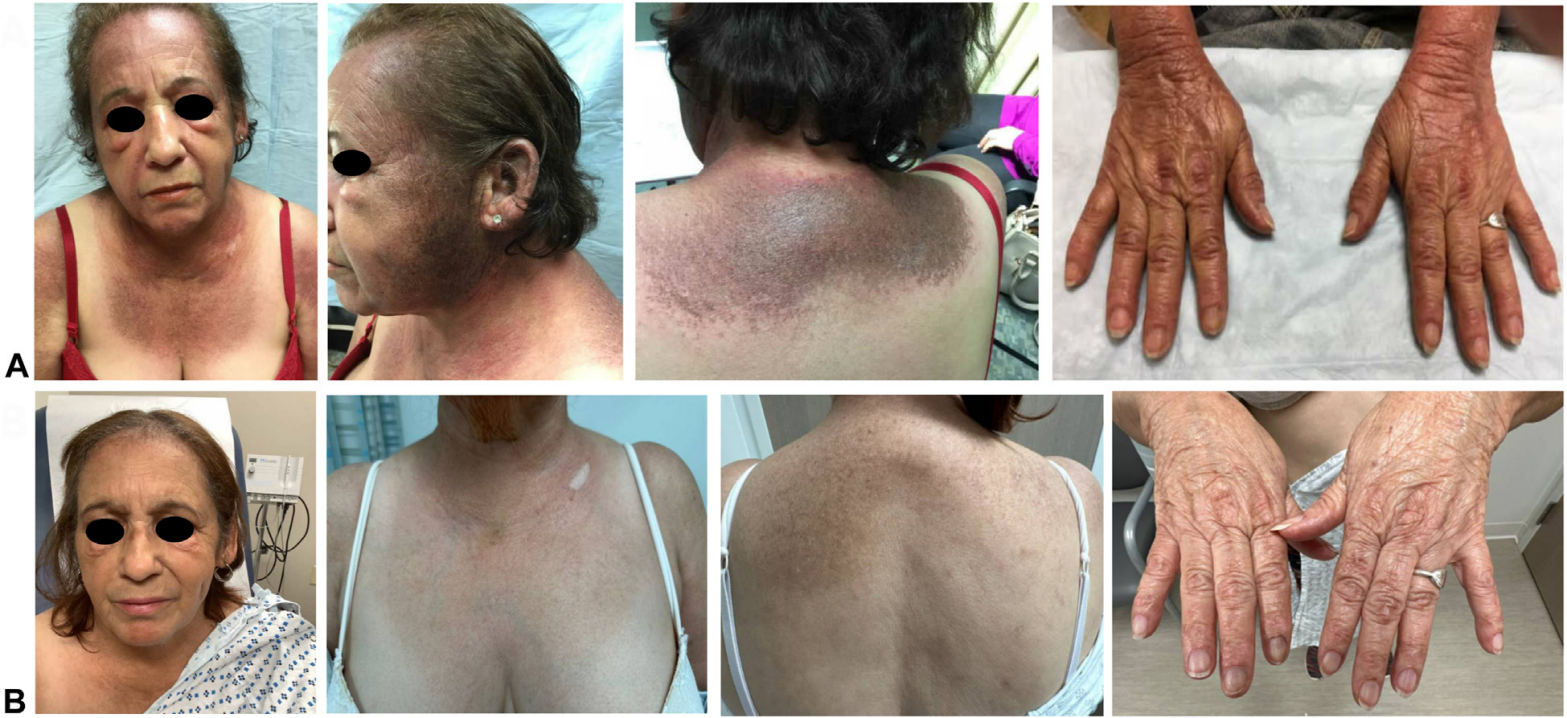
Clinical images showing dermatomyositis activity. **A,** Evidence of heliotrope rash, Gottron's erythema and papules, and shawl sign on the patient's face, upper extremities, and upper back at initial presentation. **B,** Follow-up after 10 months of treatment with upadacitinib 15 mg daily and hydroxychloroquine 200 mg twice daily.

She failed conventional treatments including hydroxychloroquine, methotrexate, mycophenolate mofetil, and intravenous immunoglobulins. Extensive cutaneous involvement persisted following 3 rounds of rituximab. In addition to hydroxychloroquine 200 mg twice daily, tofacitinib was initiated at 5 mg daily and titrated to 11 mg, which demonstrated mild improvement but lost efficacy after 1 year. She declined further intravenous immunoglobulin therapy due to prior inefficacy. Therefore, upadacitinib 15 mg daily was initiated while maintaining the hydroxychloroquine 200 mg twice daily. She started to show significant improvement within 3 months. After 12 months, there was a dramatic 32-point (Cutaneous Dermatomyositis Disease Area and Severity Index [CDASI] = 50 at the beginning of the therapy, CDASI = 18 after treatment with upadacitinib) reduction in her CDASI score, including improvement in pruritus with near-complete resolution of scalp involvement, orbital edema, poikiloderma, and Gottron’s erythema (Fig 1, *B*).

The pathophysiology of DM is poorly understood. Genome-wide association studies revealed an upregulation of interferon-inducible genes compared to healthy controls,^2^ implicating JAK inhibition as a strategy in DM management.^3^ The exact JAK pathways (JAK1, 2, or 3) that play a role in DM remain uncertain, though studies have emerged demonstrating improvements with JAK1/3 inhibitors like tofacitinib.^2^ Here, we present a case of CADM where tachyphylaxis occurred with tofacitinib but upadacitinib, a selective JAK1 inhibitor, led to a successful and sustained response. This adds to the literature suggesting JAK1 could potentially drive DM progression.^1^^,^^4^ Further research is needed to determine the optimal use of JAK inhibitors across DM subtypes, such as juvenile DM or DM with interstitial lung disease, and to evaluate their long-term safety in this population.

We hope that this case continues to provoke discussion about the course of medications that a patient should attempt prior to considering JAK inhibitors and if it should be considered as a first-line therapy.

## Conflicts of interest

None disclosed.
